# Consanguinity and perinatal medicine - the berlin perspective

**DOI:** 10.1186/1755-8166-7-S1-I51

**Published:** 2014-01-21

**Authors:** Rolf Becker

**Affiliations:** 1 Centre for Prenatal Diagnosis and Human Genetics, Berlin, Germany

## 

The present study was conducted to assess the impact of consanguinity on the prevalence of major anomalies in a prenatal study group of Berlin/Germany.

Over a time-span of 19 years (1993-2011), 34,900 fetuses were examined by prenatal sonography. In 659 cases (1.9%) the parents were consanguineous, with 45.2% related as first cousins (*F* = 0.0625) and 54.8% beyond first cousins (*F*< 0.0625). Detailed information on the pregnancy outcome of 31,145 fetuses was retrieved either through direct report by families or by active inquiry of patients or their physicians, 555 of these fetuses (1.8%) had consanguineous parentage.

The prevalence of major anomalies among fetuses with non-consanguineous parents was 2.8% (863/30,590). By comparison, in the sub-group of fetuses with consanguineous parentage the prevalence was 11.0% (61/555 fetuses). Within the consanguineous sub-group a causal association between fetal anomaly and consanguinity was assessed as probable in 6.5% (36/555) of cases, as possible in a further 3.4% (19/555) of cases, and as improbable in 1.1% (6/555) of the diagnosed anomalies.

The data indicate that the prevalence of major fetal anomalies associated with consanguinity was approximately eight percentage points higher than in non-consanguineous offspring. As a proportion of these anomalies result either in intrauterine death or medical termination of pregnancy, the prevalence of consanguinity-associated defects diagnosed post-birth is equivalently lower, thus under-estimating the overall adverse effect of intra-familial marriage on fetal and neonatal well-being.

